# Personalized Blood Glucose Forecasting from Limited CGM Data Using Incrementally Retrained LSTM

**DOI:** 10.1109/TBME.2024.3494732

**Published:** 2025-03-21

**Authors:** Yiheng Shen, Samantha Kleinberg

**Affiliations:** Department of Computer Science, Stevens Institute of Technology, Hoboken, NJ 07030, USA

**Keywords:** Blood glucose forecasting, glucose variability, incremental training, cold start forecasting

## Abstract

For people with Type 1 diabetes (T1D), accurate blood glucose (BG) forecasting is crucial for the effective delivery of insulin by Artificial Pancreas (AP) systems. Deep learning frameworks like Long Short-Term-Memory (LSTM) have been widely used to predict BG using continuous glucose monitor (CGM) data. However, these methods usually require large amounts of training data for personalized forecasts. Moreover, individuals with diabetes exhibit diverse glucose variability (GV), resulting in varying forecast accuracy. To address these limitations, we propose a novel deep learning framework: Incrementally Retrained Stacked LSTM (IS-LSTM). This approach gradually adapts to individuals’ data and employs parameter-transfer for efficiency. We compare our method to three benchmarks using two CGM datasets from individuals with T1D: OpenAPS and Replace-BG. On both datasets, our approach significantly reduces root mean square error compared to the state of the art (Stacked LSTM): from 14.55 to 10.23 mg/dL (OpenAPS) and 17.15 to 13.41 mg/dL (Replace-BG) at 30-minute Prediction Horizon (PH). Clarke error grid analysis demonstrates clinical feasibility with at least 98.81% and 97.25% of predictions within the clinically safe zone at 30- and 60-minute PHs. Further, we demonstrate the effectiveness of our method in cold-start scenarios, which helps new CGM users obtain accurate predictions.

## I. Introduction

TYPE 1 diabetes (T1D) is a lifelong disease characterized by a lack of insulin production. Individuals with T1D rely on exogenous insulin to regulate their blood glucose (BG) levels [1] and maintain health. Accurate insulin dosing is critical for individuals with T1D as keeping BG levels in a healthy range can prevent severe complications of T1D, such as kidney disease [2] and stroke [3]. However, the frequent daily decisions required to manage BG levels creates a significant burden on people with T1D [4]. Continuous glucose monitors (CGM) [5], which measure glucose in fluid between cells, have advanced the self-management of T1D by enabling frequent insight (e.g., every 5 minutes) into glucose without the need for fingersticks. CGMs are key to artificial pancreas (AP) systems [6], which use CGM data to automatically determine insulin needs and provide instructions to an insulin pump. Accurate BG prediction is thus essential for estimating insulin dosing in AP systems [7] and for taking preventative actions to avoid glycemic excursions [8].

Deep learning has been widely used for BG forecasting on both in silico and in vivo data [9]. Since prior work shows significant performance gaps between these data types for BG forecasting [10] [11], we focus our discussion on methods tested on in vivo data. Although methods such as Single Long Short-Term Memory (LSTM) [12] and CNN-LSTM [13] have become increasingly accurate on average, there is wide variation in individual-level performance. For instance, Hameed and Kleinberg reported a worst case Root Mean Square Error (RMSE) of BG prediction for an individual of 29.42 mg/dL, more than double their average RMSE of 14.53 mg/dL [12]. On another large in vivo dataset, Replace-BG [14], CNN-LSTM had an average RMSE of 14.04 mg/dL at the 30 minute-prediction horizon (PH), with a standard deviation (SD) of 4.47 [13]. One reason for significant individual differences in forecasting accuracy is that algorithms may not learn individual glucose patterns accurately without large amounts of personal data, and this may be particularly problematic for individuals with high glucose variability (GV) [15]. Thus, there is still a need for methods that work equally well for all individuals, regardless of their GV. Personalized BG prediction models can be more reliable than a single model, as they can adapt to each person’s glucose dynamics [16] [11]. However training deep learning models from scratch for new CGM users with minimal data may not yield accurate predictions [12], [13] , as deep learning relies on large amounts of data [17], [18]. Transfer learning can partially address this by using population data for training and limited personal data for fine tuning [19].However, that approach did not account for different GV profiles, which may have led to high SD (mean RMSE 18.2±7.71 for a 30-minute PH). Thus there is still a need for improved cold-start personalized forecasting.

We introduce a personalized BG forecasting approach that can be used with limited data and for individuals with a range of GV. We propose Incrementally Retrained Stacked LSTM (IS-LSTM), a method that incrementally learns each individual’s BG patterns from a stream of CGM data. Parameter transfer [20] reduces training time while maintaining accuracy. We incorporate GV into the algorithm by assigning different numbers of initial training days in the first subset of input. Further, we use pre-trained models derived from the stacked LSTM incrementally retrained on extensive data for cold start BG forecasting. These models progressively adapt to the unique BG patterns of individuals, resulting in improved accuracy compared to training from scratch, which aids new CGM users in obtaining accurate BG forecasts. Additionally, the pre-trained models enhance the generalizability of our method to individuals new to CGM and researchers without access to massive historical training data.

## II Related Work

Both statistical and machine learning (ML) algorithms have been proposed for BG forecasting at a PH of 15-60 minutes [21]. We summarize mean RMSE and SD at 30- and 60-minute PHs for works using in vivo data in Table I.

Examples of statistical methods include the auto-regressive (AR) model [22], auto-regressive with output correction (cARX) [23], and auto-regressive integrated moving average (ARIMA) [24]. However these approaches assume linearity and regularity in data, which does not hold for BG data [25], and may result in accumulating errors over time [26]. Classical ML methods include Random Forest (RF), Regression Trees [24], and Support Vector Regression (SVR) [24]. A limitation of both approaches is their requirement on engineered feature extraction from the input data, which depends on domain expertise and signal processing knowledge [16].

Recently, deep learning models have been adopted for BG forecasting. Li et al. [27] proposed Convolutional Recurrent Neural Network (CRNN), where the neural network includes a multi-layer CNN intended to capture the patterns of the multi-dimensional time series data followed by a modified RNN that is used to analyze the previous sequential data and predict BG levels. CRNN reached a mean RMSE of 9.38±0.71 mg/dL at a 30-minute PH and 18.87 ±2.25 mg/dL at a 60-minute PH on simulated data, but the prediction error increased significantly when applied to real world CGM data of 10 T1D subjects (21.07±2.35 mg/dL for 30-minute PH and 33.27±4.79 mg/dL for 60-minute PH). Zhu et al. used a Fast-adaptive and Confident Neural Network (FCNN) [28] for personalized BG prediction on the OhioT1DM dataset [29] consisting of 12 people with T1D and reached an average RMSE of 18.64±2.4 mg/dL and 31.07±3.62 mg/dL at a 30- and 60-minute PH respectively. This work aimed to solve the cold start BG forecasting problem (forecasting with little or no historical data) by enabling fast adaptation with limited data, but weeks or months of training data are required to achieve the reported accuracy. Zhu et al. subsequently implemented edge-based temporal fusion transformer (E-TFT) [30] on the same dataset, with similar accuracy. Xie and Wang [31] compared the performance of several machine learning models versus classic AR with Exogenous inputs (ARX) model on 6 people with T1D, and found that Temporal Convolution Network (TCN) proposed by Bai et al. [32] was more robust than ARX. Rabby et al. presented a stacked LSTM-based deep RNN model [33] for accurate and reliable BG prediction, with engineered features using carbohydrate, insulin, heart rate, and step counts, using data from 6 people with T1D [29]. They also found that step count can be used to improve BG prediction accuracy. Seo et al. proposed a CNN framework trained on population data and fine-tuned on personal data collected from 29 T1D subjects [19]. Their fine-tuned CNNs obtained better accuracy than the CNNs trained from scratch. Jaoli and Cescon introduced CNN-LSTM [13], a hybrid deep learning model that improved RMSE at both 30- and 60-minute PH on a large dataset [14]. They achieved on average 98.73±2.52% and 96.53±3.24% of prediction values falling within the accurate zones of the Clarke error grid analysis (EGA) for 30- and 60-minute PH. Lastly, Hameed and Kleinberg [12] compared a set of BG forecasting methods, including Linear Regression, Random Forest, Support Vector Regression, auto-regressive moving average (ARMA), RNN, and LSTM on the OhioT1DM dataset of 12 subjects and [29] and Open Artificial Pancreas System (OpenAPS) dataset [34] of 55 individuals, and found that single LSTM obtained the best accuracy in terms of mean RMSE (14.53 mg/dL) on OpenAPS for a 30-minute PH and RNN performed the best on the OhioT1DM (19.22 mg/dL for 30-minute PH). They also observed that adding more information about meals and insulin can degrade the accuracy of forecasting.

While these frameworks have advanced BG forecasting, performance can vary significantly across subjects. For instance, fine-tuned CNN achieved an average RMSE of 17.8 mg/dL at 30-minute PH, but with a high SD of 7.73 mg/dL [19]. Similarly, a single LSTM [12] reached an average RMSE of 14.53 mg/dL on the OpenAPS [34] dataset, yet the worst RMSE for an individual was 29.03 mg/dL. One reason for the difference between mean and worst-case performance is that algorithms may not be able to learn BG patterns for people with high GV accurately without large amounts of data [15]. Using LSTM and RF for forecasting, Mosquera-Lopez and Jacobs found that higher GV is strongly associated with lower forecast accuracy [15]. Adaptive models partially address challenges due to nonstationarity by adjusting their parameters online based on incoming data. Examples include the online residual compensation network-ARMA (RCN-ARMA) [35] and the sparsification criteria kernel recursive least-squares (SC-KRLS) algorithm [36]. While these adaptive models can quickly adapt to changes in glucose dynamics [37], in prior work they have lower accuracy compared to deep learning models, which leverage more complex structures. For instance, RCN-ARMA reported an average RMSE of 20.03 and 34.89 mg/dL at 30- and 60- minute PH respectively, compared to 18.57 and 30.32 mg/dL achieved by Two layer Stacked LSTM at the same PHs on the OhioT1DM dataset [33], [35]. We propose that another solution is incremental training. Although the model is not updated at every time step, incremental algorithms can continuously learn and adjust as samples are added [38]. Thus they may adapt to high GV without requiring a large amount of data from the beginning, while still maintaining the high accuracy characteristic of deep learning models.

## III. Methods

We now introduce our deep learning framework for BG forecasting. To improve individual prediction accuracy, we incrementally retrain a two-layer Stacked LSTM on continuous input of individual CGM data.

### A. Stacked LSTM

**Fig. 1.**
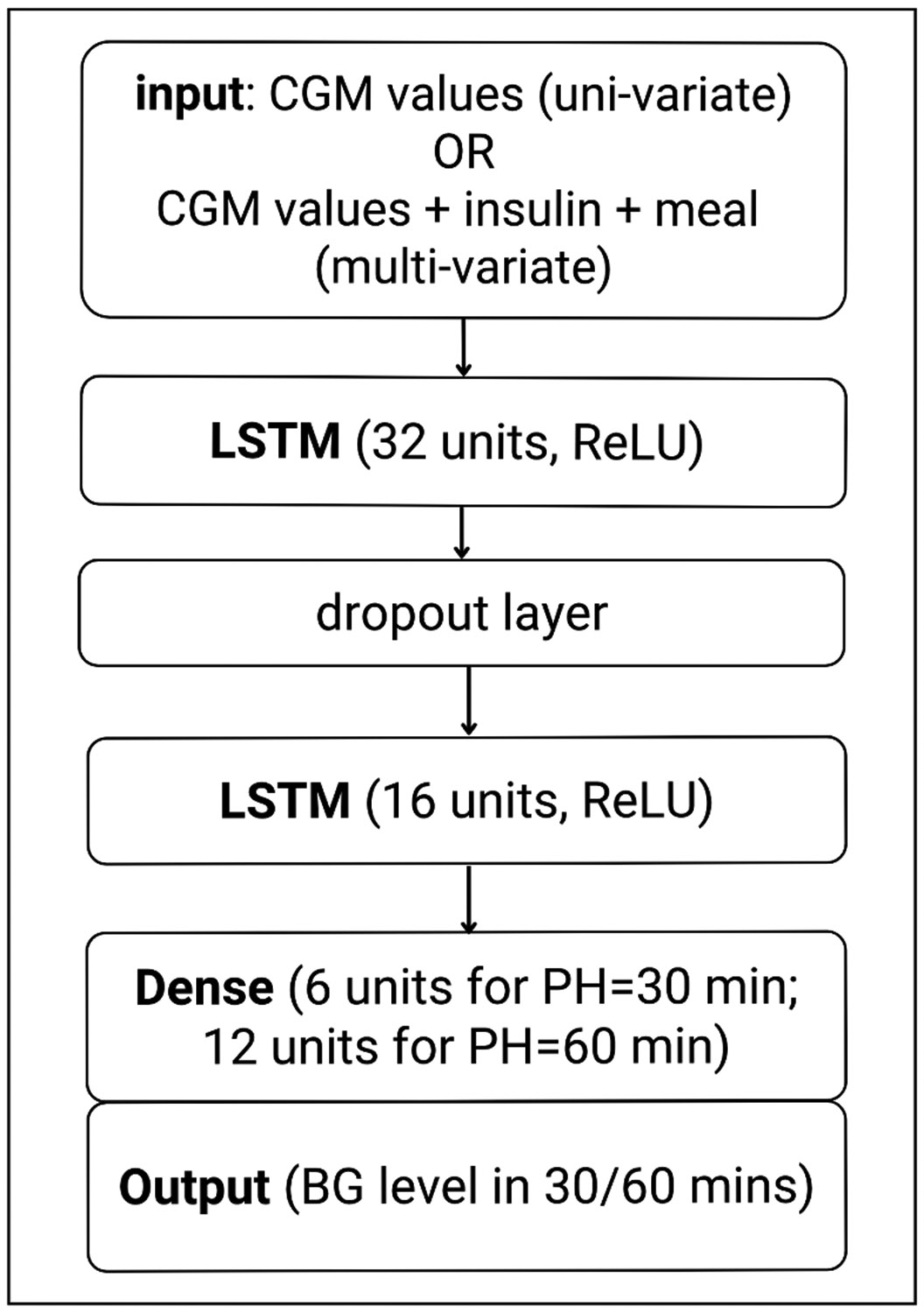
Two layered stacked LSTM model architecture.

We formulate BG prediction as a supervised learning task, where the target is to predict an individual’s BG levels at a PH (e.g., 30 or 60 minutes) given a history window of 2 hours of CGM data in a univariate or multivariate setting. For univariate BG forecasting, only CGM values are used as input. For multivariate BG forecasting, we use insulin (basal and bolus rates) and carbohydrate intake along with CGM values as inputs. We build a stacked LSTM network, which contains one LSTM layer of 32 units with the rectified linear unit (ReLU) [39] activation function, a dropout layer (dropout rate = 0.2) which is intended to reduce over-fitting [40], another LSTM layer of 16 units with activation function ReLU, and an output layer that has 6 units for a 30-minute PH or 12 units for 60-minute PH. These parameters are selected according to the results of hyper-parameter tuning using KerasTuner [41] on a subset of training data from the two datasets used in our experiments. The optimization criterion is the minimization of RMSE on the test data. The stacking of the LSTM layers increases the model’s depth, potentially enhancing its ability to abstract information and improve generalizability to previously unseen data [42]. Fig. 1 shows the high-level architecture of the stacked LSTM.

### B. Incrementally Retrained Stacked LSTM with Parameter Transfer

**Fig. 2.**
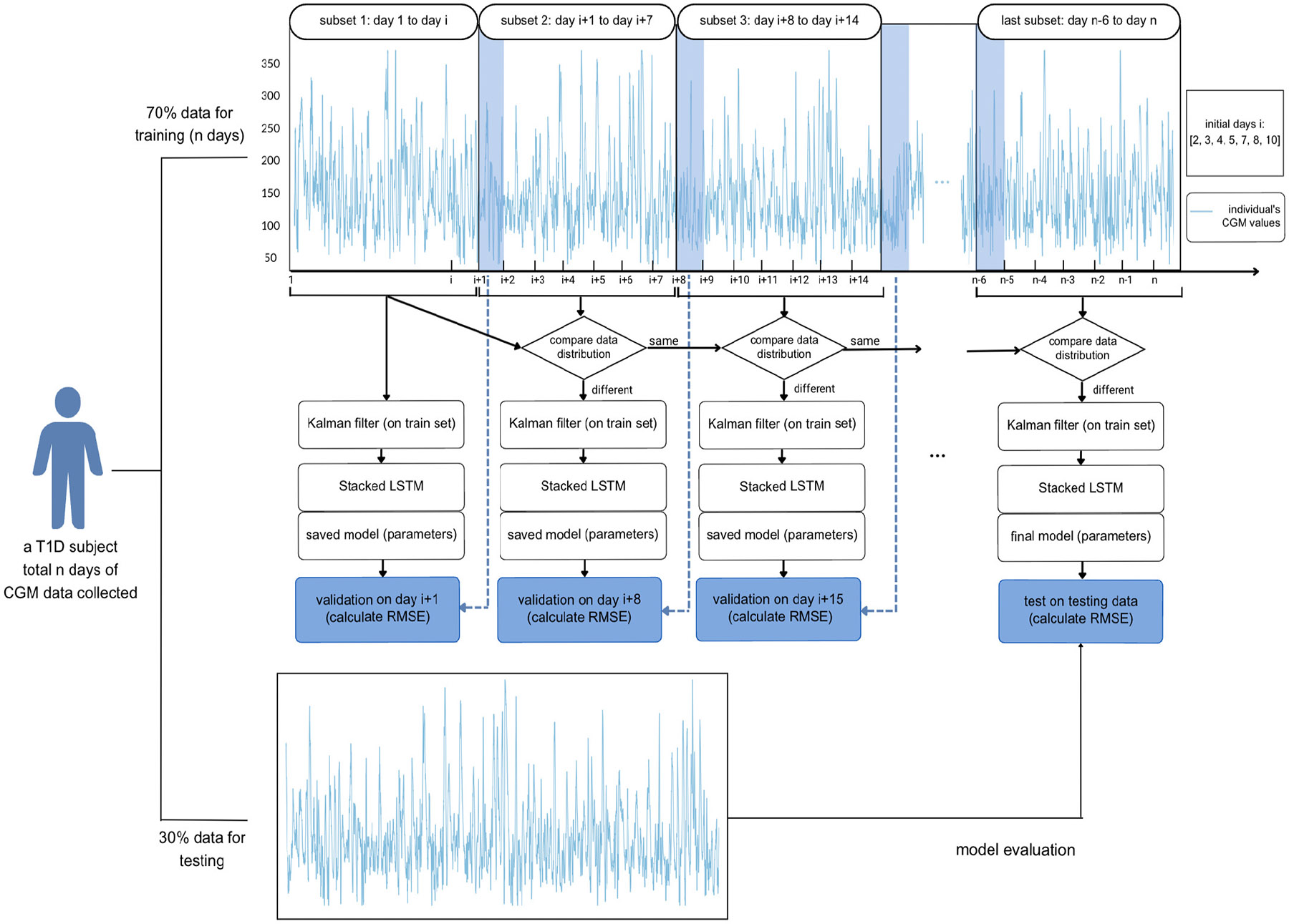
Incrementally Retrained Stacked LSTM with Parameter transfer architecture.

We incrementally retrain the stacked LSTM on continuous subsets of CGM values to improve the accuracy of personalized BG forecasting. The stacked LSTM is trained on an individual’s first subset (containing i days of data), and validated on the first day of data from the second subset ($i+1^{\mathrm{th}}$ day) by computing the RMSE between predicted and recorded CGM values. From the second to the last subsets (each containing 7 days of data), we compare the distribution of the current and previous data subsets by two-sample t-tests. If the distributions differ significantly (p≤0.05), training is repeated on the current subset, and thus model parameters are updated. If the distributions of the current and previous subsets do not differ significantly, training is skipped and the previous model parameters are used until the next iteration. The parameter transfer is designed to save training time by skipping training on data subsets with same distributions. During the incremental retraining process, the result of training on each subset is validated on the first day’s data of the subsequent subset. To restate, the training outcome of the $m^{\mathrm{th}}$ subset is validated using the first day’s data from the $m+1^{\mathrm{th}}$ subset. Fig. 2 shows an overview of the personalized IS-LSTM model parameter-transfer structure for univariate training. The dominant time complexity of univariate IS-LSTM is $O(E\times N\times T\times h_{\max}^{2})$, where E is the number of epochs of trained which could be different at different retraining stages due to early stopping, N the number of subsets trained on, T is the sequence length, and $h_{\max}$ is the largest number of hidden units. Skipping retraining on subsets with similar distributions means fewer epochs and subsets need to be trained on.

### C. Glucose Variability Measurement

We measure subjects’ GV with 8 clinically validated GV metrics [43], including high blood glucose index (HBGI), low blood glucose index (LBGI), glycemic variability metric (J_index), percentage of time in range (TIR), interday coefficient of variation (interday CV, %), mean intraday CV, interday standard deviation (SD, mg/dL), and glycemic management indicator (GMI). We divided the subjects in both datasets into low and high GV using a threshold of %CV=36% , as prior work identified this as a suitable threshold to distinguish high from low GV in people with diabetes [44].

Table II presents the mean of each GV metric for both groups. We demonstrate that 36% is a suitable CV threshold for distinguishing between low and high GV subjects, as most of the GV metrics are significantly lower for the low GV group than the high GV group. It has been found previously that more variable glucose is associated with lower forecast accuracy independent of the prediction algorithms [15] [45]. Therefore, the significant variations in the GV metrics between the high and low GV groups suggests that predictions may require different model configurations for each group.

## IV. Experiments

We aim to evaluate our IS-LSTM method with Parameter Transfer for the glucose forecasting across individuals with different amounts of personal data collected. This evaluation covers two scenarios: 1) training from scratch for individuals with months of data collected and 2) cold-start forecasting for those new to CGM. First, we compare our method against online RCN-ARMA [35] and three recent deep learning benchmarks that reached an average RMSE below 15 mg/dL at a 30- minute PH. We re-implemented these methods on two large scale T1D datasets, using individual data in both univariate and multivariate setups. Second, to test the models’ ability on cold-start forecasting we leverage the individual models generated through the incremental retraining by employing them as pre-trained models.

### A. Datasets

#### 1) OpenAPS Data Commons:

The OpenAPS dataset includes data from 183 people with T1D who donated their data for research use through the Open Artificial Pancreas System (OpenAPS) [34] project. The dataset consists of CGM data recorded at 5 min intervals, insulin basal rate and bolus events, and self-reported carbohydrate intake in grams. We used a subset of the OpenAPS dataset, only including participants who have more than 50 days of data so that there are at least 5 iterations in the incremental training of stacked LSTM. This resulted in a final dataset of 126 participants with a mean of 403±342 days of data (median 276 days).

#### 2) Replace-BG dataset:

The Replace-BG dataset was collected during a randomized controlled trial testing the use of CGM without BG confirmations [14]. It has 226 participants with a mean of 234.3±36 days of data (median 257.4 days) collected, including CGM, finger-stick BG, basal rates, bolus doses, and other self-reported information such as age, race, and annual household income. All subjects in the Replace-BG dataset have more than 50 days of data recorded.

### B. Data Processing

#### 1) Data Cleaning:

We use four variables from the OpenAPS dataset: CGM values (mg/dL), insulin basal rates, insulin bolus values, and self-reported carbohydrates consumed during a meal (g). We use three variables from Replace-BG: CGM values (mg/dL), insulin basal rates, and insulin bolus values. We excluded CGM values below 15 mg/dL as biologically implausible [12]. Next, to address missing CGM values in training data, we used linear interpolation, a widely used method for filling gaps in time series data. [46]. We imputed values for gaps less than 30 minutes long using (1).


(1) $$x_{t}=\frac{x_{t-1}-x_{t+1}}{t_{-1}-t_{+1}}(t-t_{+1})+x_{t+1}$$

On test data we used first-order extrapolation to avoid using future data. For basal insulin, we propagate the rate forward until a change is recorded. We evenly distributed bolus rates across the specified duration. When no values were recorded, both bolus rates and carbohydrate intake were set to 0.

#### 2) Kalman Filter on Training Data:

**Fig. 3.**
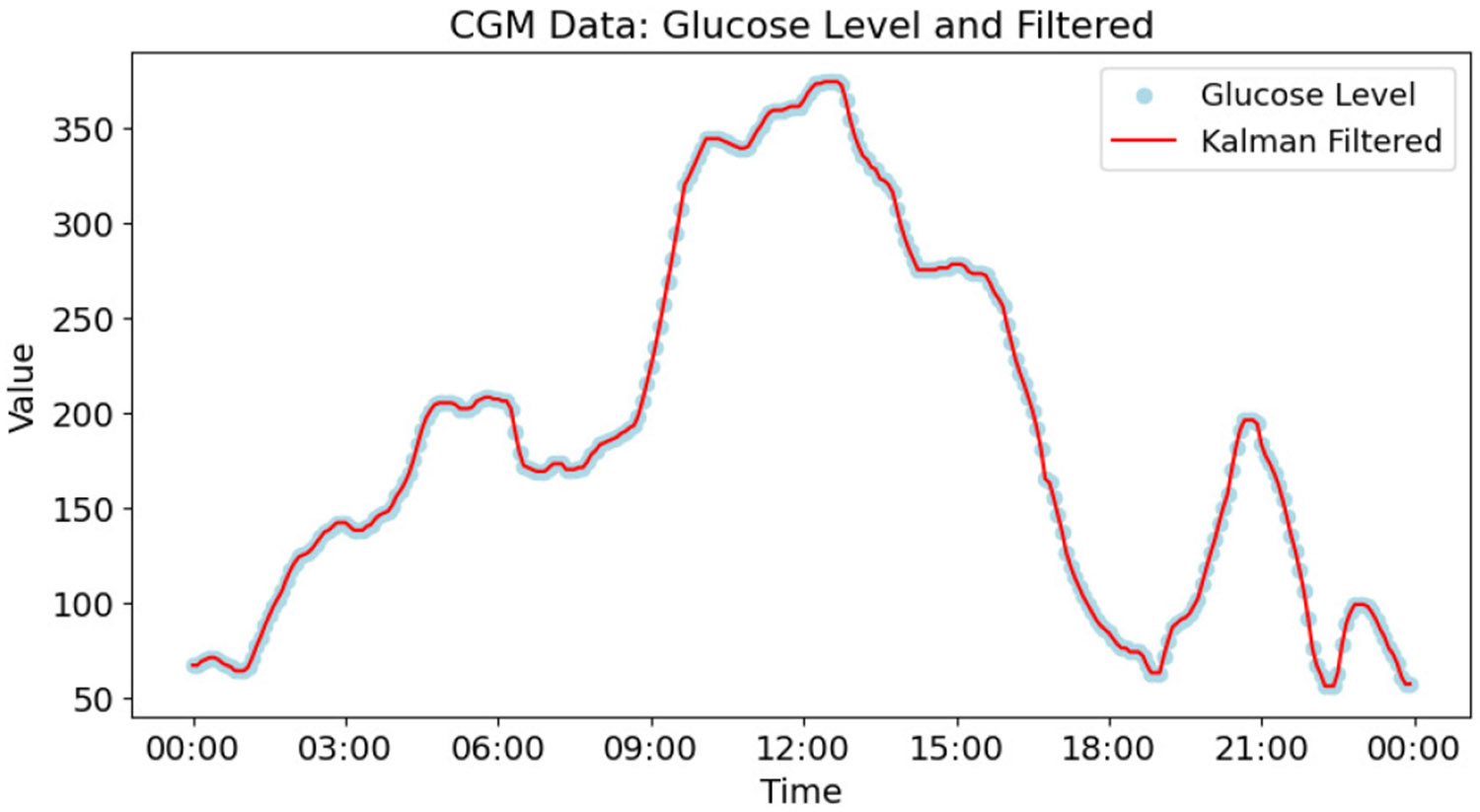
Kalman Filter smoothed CGM values and raw CGM values over 24 hours for one individual.

Finally, we applied a Kalman Filter [47] to smooth the CGM values in the training data, which is intended to improve the performance of LSTM networks [48]. Fig.3 shows a sample of raw CGM data and the CGM values smoothed by the Kalman Filter on a 24 hour window. We do not apply Kalman Filtering on validation or testing data.

### C. Incrementally Retrained Stacked LSTM

We build a personalized IS-LSTM model for each subject using their individual data. We split each subject’s data into non-overlapping subsets in chronological order, namely $D_{k}$, k=1,2,…,K. The first subset contains i days of data, where i is in [2, 3, 4, 5, 7, 8, 10], and the remaining subsets each contain 7 days of data. We apply the incremental retraining process described above to train models for each subject using their own data and record the RMSE obtained from each validation step. All individual models were trained for 50 epochs in each iteration, with an early stopping of a patience parameter set to 5. Retraining on each subset is terminated when there is no improvement on the validation loss (measured by Mean Square Error) for 5 consecutive epochs.

Using the processed data, we compare against several recent deep learning baselines that reached an RMSE below 15 mg/dL at a 30-minute PH for BG forecasting, including Single LSTM [12], Stacked LSTM [33], and CNN-LSTM [13]. We also implement our IS-LSTM without parameter transfer to evaluate each component of our method and compare training time. Each of the models has 2 settings: univariate prediction and multivariate prediction. In both settings two hours (120 minutes) of historical data were used to predict glucose at a 30- and 60-minute PH. In univariate prediction the input is only CGM values, while in multivariate prediction the inputs are CGM values, insulin basal rate and bolus values, and carbohydrates. In both settings, we experiment with the number of initial days in the first subset, varying them within the set [2, 3, 4, 5, 7, 8, 10]. Each experiment was repeated 5 times and average RMSE and SD were calculated across all the trials for each participant. For each subject, we train and validate all methods on the first 70% of the data subsets in chronological order, and test the model on the last 30% of the time series. The training and validation process is described in III-B. We calculate postprandial glucose forecasting accuracy by averaging the RMSE during the two hours following self-reported meals on the testing dataset [49].

### D. Cold Start Forecasting with Pre-trained Population Models

In our first experiment we trained on 70% of the data, but this can lead to a long delay in being able to use the results. Thus we next examined whether pre-trained models can be used to improve results when there is no past training data. For OpenAPS we have a total of 126 stacked LSTM models (one per person) from the first experiment. Of these we used 20 models that were trained on individual data from people who each had more than 1000 days of training data. We aim to select the best model for each of the 126 subjects. To do this we applied each of the 20 models to forecast BG for the person’s first day of data and selected the best one based on the average RMSE tested on day 1 data. This replicates a real-world situation where a person may want to select from available models without waiting to collect a significant amount of their own data. From each subject’s data from day 2 to day 50, we continue the incremental training on the selected model with a 1-day update window and test on the next day of data, replicating the case where someone starts using a CGM and has no past BG data. For Replace-BG we selected 32 models that were trained on more than 400 days of individual data as the pre-trained models, and implemented the same procedure for the 226 Replace-BG subjects.

Following the initial daily training on 50 days of data, each model was then incrementally retrained on the participant’s remaining data, with a 7-day update window. During this process, the model was validated on data from the first day of the next subset as described in III-B, allowing it to adapt and capture the participant’s weekly BG profiles. Each cold-start experiment was repeated 5 times.

### E. Evaluation Metrics

Following previous work, we use RMSE, given by (2), as the primary evaluation metric. Here $g_{t}$ and ${\hat{g}}_{t}$ are the actual and estimated glucose values.


(2) $$RMSE=\sqrt{\frac{1}{T}\Sigma_{t=1}^{T}{\left(g_{t}-{\hat{g}}_{t}\right)}^{2}}$$

We also adopt the widely used EGA [50] to measure the clinical validity of the proposed method. The EGA scatter plots depict the alignment of recorded and predicted CGM values into five significant zones. Zones A and B are considered clinically safe, whereas predictions falling within zones C, D, and E could result in dangerously incorrect treatment due to mistaking low for high glucose (or vice versa) [51]. Both RMSE and Clarke EGA are evaluated on the held out test data.

## V. Results

We first present results comparing our IS-LSTM to baselines at a 30- and 60-minute PH, then analyze how GV relates to forecast accuracy, and finally present results of using pre-trained models for cold start forecasting.

### A. Incrementally Retrained Stacked LSTM

Tables III and V report results for univariate and multivariate forecasting of our method against other benchmarks, averaged across across all 126 OpenAPS and 226 Replace-BG participants. We also report the average training time for each model. The data distributions of RMSE in each scenario passed the Lilliefors test for Gaussianity.

#### 1) Univariate forecasting:

As shown in Table III, on both the OpenAPS dataset and the Replace-BG dataset IS-LSTM with parameter transfer had the lowest RMSE at 30-minute PH, while the same model without parameter transfer performed best at a 60-minute PH. However, a t-test showed no statistically significance differences between IS-LSTM with and without parameter transfer for both PHs (p=0.59 for 30-minute PH and p=0.31 for 60-minute PH). Table IV shows the average RMSE during incremental retraining of univariate IS-LSTM with and without parameter transfer for 7 initial days of data and 80 weeks total. It also shows how many subjects have that amount of data at each point. There are no statistically significant differences in IS-LSTM with and without parameter transfer at any training stages.

#### 2) Multivariate forecasting:

The lowest RMSE in the multivariate scenario is reached by our approach, IS-LSTM with parameter transfer, at both 30- and 60-minute PH. Results of multivariate forecasting are reported in V. Using a t-test to compare results, RMSE for all multivariate models was significantly higher than that of univariate models at the same PHs (all p ≤ 0.05) for both datasets.

We also employ t-tests to test for differences between the results of our methods and other baselines. The RMSE of IS-LSTM with parameter transfer is significantly lower than that of the leading baseline stacked LSTM (p ≤ 0.01) for both datasets. Compared with adaptive RCN-ARMA model, deep learning methods had significantly lower RMSE and were faster to train. IS-LSTM with and without parameter transfer are not significantly different for both PHs (p=0.39 for 30-minute PH and p=0.18 for 60-minute PH), showing that our approach has the same accuracy at lower computational cost.

Fig. 6 shows the rate of change in mean RMSE during retraining and validation for two settings of initial days at a 30-minute PH. We annotate the mean RMSE±SD at around 10, 20, 50, 100, 200, 300, and 365 days of total input. The shaded areas represent the SD of RMSE.

### B. Clarke Error Grid Analysis

**Fig. 4.**
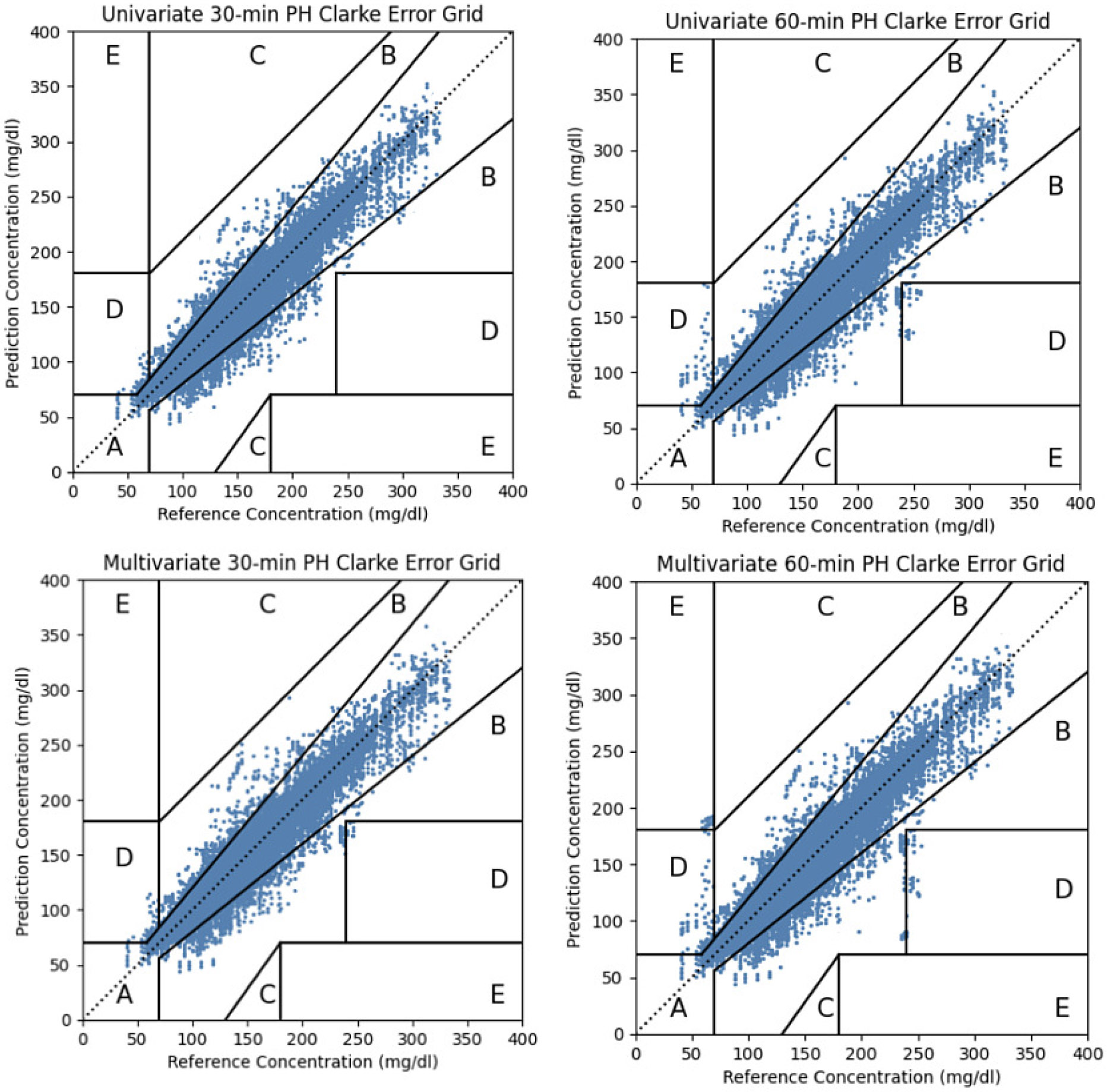
Clarke EGA for BG predictions made by the univariate and multivariate IS-LSTM for PH of 30 minutes (left) and 60 minutes (right) for one representative OpenAPS subject.

The Clarke EGA shows that for OpenAPS data predictions of the univariate IS-LSTM with parameter transfer are clinically safe with 99.78% of all predictions in zone A or zone B for a 30-minute PH and 98.49% for a 60-minute PH. There are on average 0.041% points falling in D or E regions at 30-minute PH and 0.17% in the D or E regions at a 60-minute PH. The multivariate IS-LSTM has 99.67% of all predictions in zone A or zone B for 30-minute PH and 98.05% for 60-minute PH. The best baseline, univariate Stacked LSTM, achieves 95.71% and 92.83% of all predictions within Zone A or Zone B for 30- and 60-minute PH. For the Replace-BG dataset the univariate IS-LSTM with parameter transfer predicts glucose levels clinically safely with 98.81% of predictions in zone A or zone B for 30-minute PH and 97.25% for 60-minute PH. On average 0.059% points are in D or E regions at 30-minute PH and 0.24% are in the D or E regions at 60-minute PH. The multivariate IS-LSTM has 98.67% predictions within zone A or zone B for 30-minute PH and 97.15% for 60-minute PH. In comparison, univariate Stacked LSTM reaches 93.92% and 91.54% of all predictions falling in Zone A or Zone B. Fig. 4 shows an example for one representative OpenAPS participant who has 208 days of data for testing at both PHs.

### C. Postprandial Forecasting Accuracy

The results of postprandial (2 hours after self-reported meal start) forecasting on the OpenAPS dataset are shown in Table VI. There was an average of 487 eating occasions (median 193) per person, with mean size of 27.26 grams of carbohydrates. The best average postprandial BG forecasting accuracy in terms of RMSE across all subjects was 11.49±1.6 mg/dL at 30-minute PH and 17.61±2.3 mg/dL at 60-minute PH achieved by univariate IS-LSTM. Overall, univariate forecasting achieved significantly better accuracy compared to multivariate forecasting using all models. The accuracy of IS-LSTM with and without parameter transfer were not statistically significantly different and they are significantly better than other methods. IS-LSTM’s accuracy of postprandial BG forecasting was on average 1.4 mg/dL higher than the overall average accuracy (all p≤ 0.05).

### D. Glucose Variability and Forecast Accuracy

**Fig. 5.**
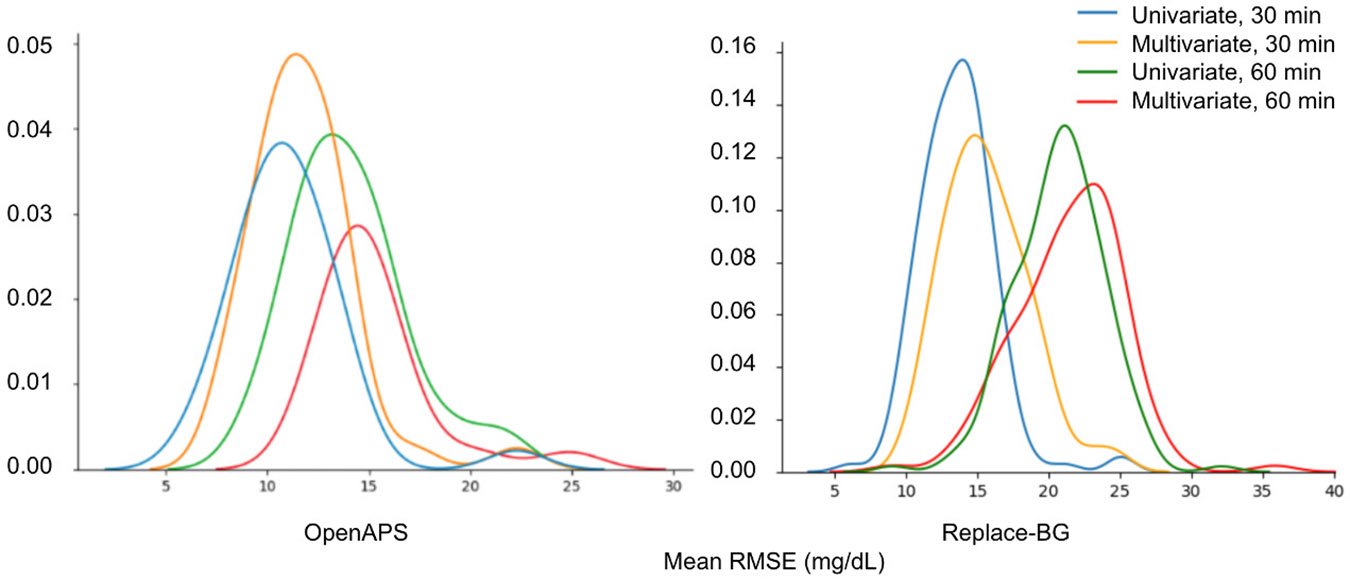
Probability density plot for BG predictions of univariate and multivariate IS-LSTM with parameter-transfer at 30- and 60-minute PHs of OpenAPS and Replace-BG.

We next analyze the variability in the accuracy of the model across different subjects. Fig. 5 shows the probability density of average RMSE per person for different types of training for OpenAPS and Replace-BG respectively. The long tails indicate that RMSE is significantly higher for some subjects in both datasets. Examining subjects in the right tails with high RMSE we find they are similar, all belonging to the high GV group.

**Fig. 6.**
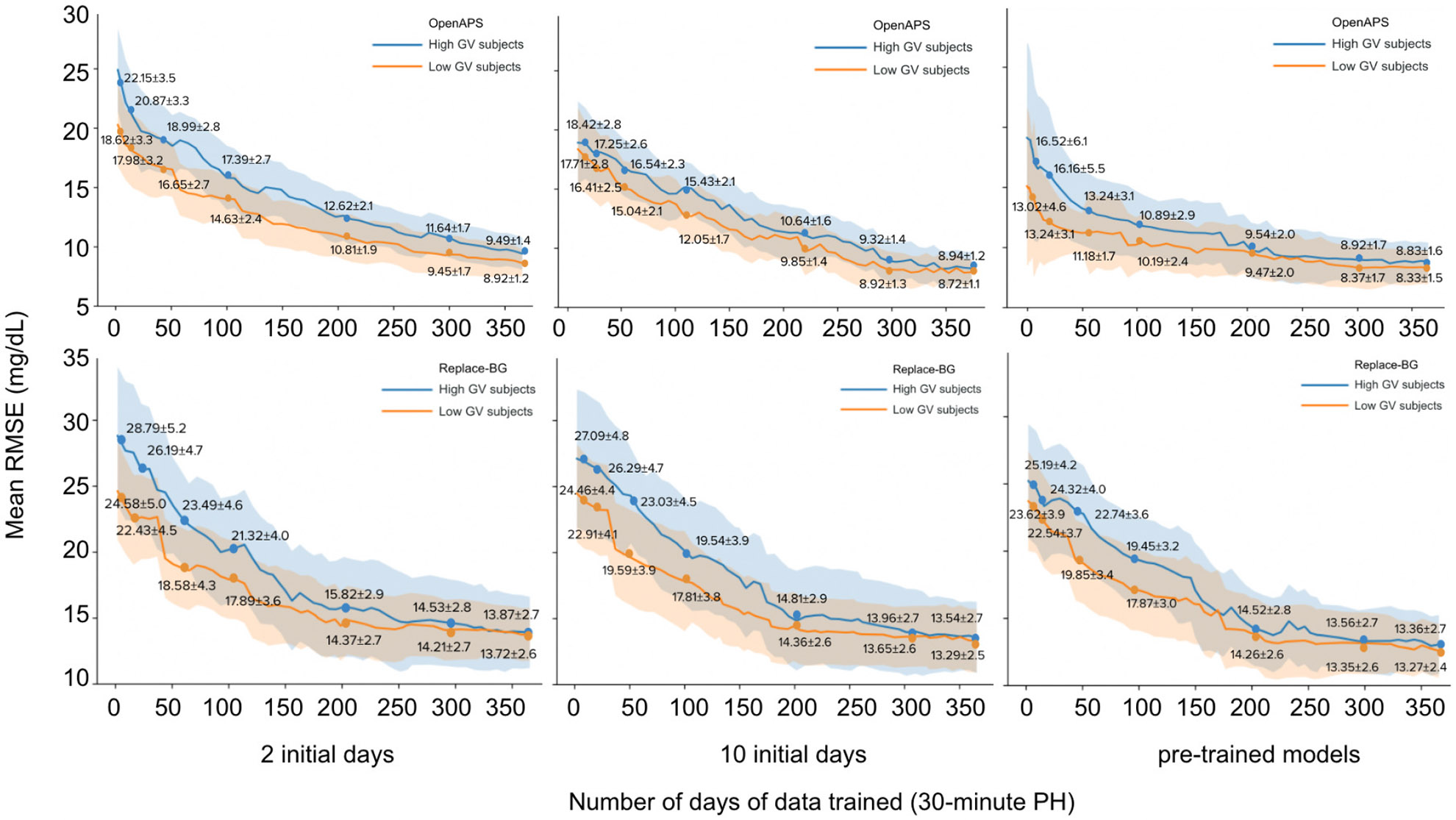
Mean RMSE of univariate IS-LSTM with parameter transfer (30-minute PH) validated on the next day CGM data across iterations of training of high GV group vs. low GV group.

Since IS-LSTM’s accuracy is lower for individuals with higher GV, we examined whether beginning with more initial training days may improve results. With higher variability, a person’s glucose patterns may be more difficult to capture with LSTM, so beginning with more days may lead to a more representative sample. We thus compared the average RMSE of the high GV group and low GV group from the univariate IS-LSTM with parameter transfer model at 30-minute PH across different numbers of initial days, as shown in Table VII. without more initial days, RMSE for the high GV group decreases, while there are no significant changes for the low GV group as indicated by pairwise t-tests. When the number of initial days is below 7, the RMSE of the low GV group is significantly lower than that of the high GV group for OpenAPS subjects. Likewise, when the number of initial days is below 8, the RMSE of the low GV group is significantly lower than that in the high GV group for the Replace-BG subjects. Fig. 6 shows that IS-LSTM with 10 initial days obtained lower RMSE than IS-LSTM with 2 initial days during the first several iterations of training.

Average RMSE for IS-LSTM decreases at different rates for the high GV and low GV group during the weekly updates of incremental retraining. As shown in Fig. 6, the difference in the average RMSE of the 2 groups stays large during the first several iterations, then gradually decreases. This indicates that IS-LSTM learns the low GV group’s glucose patterns faster, but does ultimately capture the dynamics of the higher GV group, reaching an RMSE of 15.04 mg/dL after 50 days of input, and improving over time to a best average RMSE of 8.33 mg/dL on the OpenAPS dataset and 13.17 mg/dL on the Replace-BG dataset.

### E. Cold Start Forecasting with Pre-trained Population Models

We now compare RMSE from IS-LSTM with parameter transfer trained from scratch to that of cold-start using pretrained models at approximately 10, 20, 30, 40, 50, 100, 200, 300, and 365 days of total input. Results for both OpenAPS and Replace-BG are shown in Fig. 6. Both pre-trained IS-LSTM and the IS-LSTM trained from scratch have decreasing RMSE over time, as they learn each individual’s pattern and eventually converge to the same level of accuracy, with average RMSE of 8.33 mg/dL for OpenAPS and 13.17 mg/dL for Replace-BG given enough training data. However, they differ in their convergence rates. The pre-trained models converge faster during the first few iterations, suggesting that such models can enable individuals who are new to CGM or do not have historical CGM data to obtain accurate forecasts sooner.

## VI. Discussion

We find that IS-LSTM significantly reduced RMSE for BG forecasting compared to prior works. Our method achieved a mean RMSE of 10.23 mg/dL for a 30-minute PH on OpenAPS data, outperforming the best prior work, Stacked LSTM, which had a mean RMSE of 14.55 mg/dL for the same PH. On the Replace-BG data, we improved the mean RMSE of Stacked LSTM from 18.81 to 13.56 mg/dL at a 30-minute PH. Our approach’s accuracy of BG prediction after meals is demonstrated by achieving an average RMSE of 11.49 mg/dL and 17.61 mg/dL for the 30- and 60-minute PH respectively. Parameter transfer enables more efficient training by skipping training on consecutive subsets that have same distribution. Average performance with and without parameter transfer are equivalent across different PH and number of variables, yet parameter transfer on average saves 22% of training time. For people with a long history of CGM data, IS-LSTM with parameter transfer could be more efficient in terms of training time, without compromising the model’s accuracy. Saved training time also enhances the feasibility of applying our approach to larger datasets within AP systems [52]. This efficiency is crucial for real-world applications, where computational resources and time are often constrained.

Given the influence of GV on the performance of BG forecasting models [15] [45], we investigated the relationship between individuals’ GV and the accuracy of IS-LSTM models. The difference in forecast accuracy between the high and low GV groups with different number of initial days suggests different number of initial days in the models are needed. Based on our results, we find that when an individual’s GV is high, we should use 7- to 10-day’s data for the initial iteration and rest data for incremental training. If the individual’s GV is low, we can use 2-5 days of data for the initial iteration. For people with T1D who recently adopted CGM or have few days of CGM data (20 days, for example), our pre-trained IS-LSTM can help them obtain accurate and personalized BG forecasts without the need to collect many months of data before beginning forecasting. While IS-LSTM is not an adaptive model that responds to real time changes in blood glucose levels as adaptive models do [35]-[37], it can adapt to changes as it retrains on each coming week’s data.

Personalized, accurate, and efficient BG forecasting can have a substantial impact on multiple areas of T1D self-management. First, the efficacy of AP systems are improved by better glucose forecasting methods. If an AP could accurately estimate future BG in advance, reminders of insulin boluses could be sent in advance. Second, higher confidence in the AP supported by robust BG forecasting may reduce patients’ anxiety about managing their condition, which in turn can lead to an improved overall quality of life [53]. Third, glucose prediction models have been used in AP systems that improved time-in-range and other glycemic outcomes [37]. Moreover BG forecasting algorithms have found application in decision support systems for people with diabetes [54], where the support system uses BG forecasts to send users treatment recommendations and exercise risk warnings.

One limitation of our work is that like prior works we observe lower accuracy with multivariate training at both 30- and 60-minute PH. Mean RMSE for 30-minute PH is on average 1.4 mg/dL higher on the OpenAPS data when the IS-LSTM incorporates insulin basal rate, bolus values, and carbohydrates. It seems counter-intuitive that including more variables that are known to influence BG (insulin, carbohydrates) would negatively impact accuracy. However, it may be due to fewer data points for these variables (only a few meals a day compared to hundreds of BG values), unreliable or missing meal reports from OpenAPS participants [55], or the patterns of influence being more complex than can be captured by the LSTM. Similarly, while it is more challenging to predict BG further in the future, it may be more useful as it allows for more advance planning with insulin administration, as BG disturbances such as a meal could triggers a rapid glucose rise that is faster than the time needed for insulin absorption and action [7]. However, RMSE is notably higher for glucose prediction in 60 minutes, and accuracy typically decreases as PH increases [48]. Reduced accuracy after mealtimes could be attributed to significantly higher glucose levels during the 2-hour period after meals compared to before meals (with an average increase of 19.43 mg/dL across the 126 OpenAPS participants). The elevated post-meal glucose levels make prediction more challenging, especially given the lower before meal glucose values [56]. Meanwhile, the influence of bolus insulin on postprandial BG levels adds complexity to predicting post-meal glucose levels. Another limitation is that our model does not consider disturbances to glycemia, such as physical activity or illness. Further investigation is needed to fully explore the optimal use of dietary intake, physical activity, medical history, and insulin data for long-term BG forecasting.

## VII. Conclusion

We introduced IS-LSTM with Parameter Transfer for BG forecasting and showed that it outperforms the state of the art on BG forecasting on two datasets. Parameter transfer reduced training time while keeping the same level of accuracy. We demonstrated that there is a negative relationship between RMSE and GV, but showed how this can be overcome by adjusting the number of initial days for IS-LSTM. Individuals with high GV require more initial days to reach ideal model performance. Our pre-trained IS-LSTM provides accurate glucose prediction while requiring only a small amount of data, thus improving the accuracy of personalized BG forecasting for people who are new to CGM. Ultimately this work could be integrated into AP systems or used in CGM forecast displays. In future work we aim to improve accuracy at longer horizons, which may allow finer control over BG.

## Figures and Tables

**TABLE I:** Summary of previous studies on BG forecasting on real world datasets from people with T1D at 30- and 60-minute PH.

Method	Cohort	Mean RMSE±SD (mg/dL)	
		PH = 30min	PH = 60min
AR (Wang et al, 2013) [22]	10 subjects	27.7	-
cARX (Botwery et al, 2014) [23]	10 subjects	26.1	-
RCN-ARMA	6 subjects	20.03	34.89
(Ma et al., 2020) [35]	from OhioT1DM Dataset [29]		
SC-KRLS	3 subjects	18.53	-
(Yu et al., 2020)[36]			
ARIMA (Rodríguez-Rodríguez et al, 2019) [24]	25 subjects	17.31	27.60
RF (Rodríguez-Rodríguez et al, 2019) [24]	25 subjects	14.63	22.12
SVR (Rodríguez-Rodríguez et al, 2019) [24]	25 subjects	20.82	26.36
TCN (Xie and Wang, 2018) [31]	6 subjects from OhioT1DM Dataset [29]	20.54±3.36	-
CRNN (Li et al, 2020) [27]	10 subjects	21.07±2.35	33.27±4.79
Single LSTM (Hameed and Kleinberg, 2020) [12]	55 subjects from OpenAPS Dataset [34]	14.53	-
Two-layer Stacked LSTM (without Kalman Smoother) (Rabby et al, 2021) [33]	6 subjects from OhioT1DM Dataset [29]	18.57	30.32
Fine-tuned CNN (Seo et al, 2021) [19]	29 subjects	17.8±7.73	28.1±12.61
CNN-LSTM (Jaloli and Cescon, 2022) [13]	168 subjects from Replace-BG Dataset [14]	14.04±4.47	27.19±5.59
FCNN (Zhu et al, 2022) [28]	12 subjects from OhioT1DM Dataset [29]	18.64±2.60	31.07±3.62
E-TFT (Zhu et al, 2022) [30]	12 subjects from OhioT1DM Dataset [29]	19.09±2.47	32.31±3.79

*Notes.* There are in total 226 subjects in the Replace-BG dataset.

**TABLE II:** Mean GV metrics for both datasets used, comparing individuals with high and low GV

Metric	OpenAPS		Replace-BG	
	High GV (CV>36% n=61)	Low GV (CV≤36% n=65)	High GV (CV>36% n=145)	Low GV (CV≤36% n=81)
Interday SD	58.56	41.66*	65.44	51.18*
Mean intraday CV	33.19	26.76*	33.77	27.71*
Interday CV	39.53	31.49*	40.13	32.63*
TIR%	39.53	31.49*	68.05	69.01
J_index	43.10	30.56*	53.00	44.07*
HBGI	5.77	3.13*	7.71	6.02*
LBGI	1.21	1.07	1.12	0.60*
GMI	6.85	6.45*	7.21	7.06

*Notes.* A value marked by an asterisk * is statistically significantly lower than the value in the other column within the same dataset (p≤ 0.05).

**TABLE III:** Mean RMSE±SD (mg/dL) and training time for univariate glucose prediction with 30- and 60-minute PHs

Method	OpenAPS			Replace-BG		
	PH = 30 min	PH = 60 min	Average time	PH = 30 min	PH = 60 min	Average time
RCN-ARMA (Ma et al., 2020) [35]	19.65±3.8	31.78±4.8	113 min	20.93±4.2	35.97±5.3	87 min
Single LSTM (Hameed and Kleinberg, 2020) [12]	17.62±2.6	28.14±3.2	21 min	18.81±2.8	29.34±3.7	16 min
CNN-LSTM (Jaloli and Cescon, 2022) [13]	16.83±2.4	27.51±2.8	23 min	17.15±2.3	28.73±3.7	19 min
Stacked LSTM (Rabby et al, 2021) [33]	14.55±2.3	19.3±2.6	27 min	17.82±2.1	21.54±3.3	19 min
IS-LSTM without Parameter Transfer	**10.41**±**1.3**	**14.97**±**1.9**	46 min	**13.56**±**2.1**	**19.97**±**2.8**	28 min
IS-LSTM with Parameter Transfer	**10.23**±**1.3**	**15.08**±**1.8**	37 min	**13.41**±**2.0**	**19.68**±**2.8**	23 min

*Notes.* The bold values are significantly lower (p≤ 0.01) than non-bolded values in the same column.

**TABLE IV:** Mean RMSE±SD (mg/dL) of univariate IS-LSTM with and without parameter transfer with 7 initial days at 30- and 60-minute PHs on the OpenAPS dataset

weeks of data trained	IS-LSTM with parameter transfer		IS-LSTM without parameter transfer	
	PH = 30 min	PH = 60 min	PH = 30 min	PH = 60 min
1 week (126 subjects)	20.15±3.4	28.04±3.8	20.12±3.5	28.19±3.9
2 week (126 subjects)	19.37±3.3	27.68±3.6	19.06±3.3	27.44±3.7
3 week (126 subjects)	18.92±2.8	25.62±3.4	18.31±2.7	24.81±3.5
5 week (126 subjects)	17.64±2.7	24.31±3.2	17.36±2.7	24.04±3.1
7 week (126 subjects)	15.61±2.6	22.46±3.1	15.48±2.5	22.32±3.0
10 week (120 subjects)	14.88±2.4	21.13±3.0	14.69±2.4	21.04±3.0
15 week (114 subjects)	13.23±2.1	19.31±2.8	13.30±2.2	19.35±2.7
20 week(105 subjects)	12.81±1.8	18.25±2.6	12.74±1.9	18.67±2.5
30 week (95 subjects)	11.25±1.7	16.32±2.5	11.21±1.7	16.29±2.6
40 week (80 subjects)	10.53±1.3	15.06±2.0	10.63±1.3	14.97±1.9
50 week (67 subjects)	9.25±1.2	14.72±1.8	9.27±1.3	14.59±1.8
60 week (56 subjects)	9.01±1.2	14.33±1.6	9.11±1.2	14.28±1.7
70 week (47 subjects)	8.54±1.1	14.16±1.4	8.61±1.1	14.08±1.5
80 week (39 subjects)	8.31±1.0	13.38±1.2	8.41±1.0	13.27±1.3

*Notes.* The reported RMSE is the mean RMSE validated on the next day CGM data during retraining.

**TABLE V:** Mean RMSE±SD (mg/dL) for multivariate glucose prediction with 30- and 60-minute PHs on the OpenAPS dataset

Method	OpenAPS			Replace-BG		
	PH = 30 min	PH = 60 min	Average time	PH = 30 min	PH = 60 min	Average time
Single LSTM (Hameed and Kleinberg, 2020) [12]	18.27±2.9	29.86±3.7	21 min	19.82±2.8	31.04±4.1	16 min
CNN-LSTM (Jaloli and Cescon, 2022) [13]	17.46±3.0	29.18±3.5	23 min	18.70±2.5	29.64±3.9	20 min
Stacked LSTM (Rabby et al, 2021) [33]	15.61±2.6	21.34±3.1	27 min	17.46±2.1	23.03±3.4	19 min
IS-LSTM without Parameter Transfer	**11.74**±**1.7**	**15.96**±**2.0**	46 min	**14.17**±**2.0**	**21.92**±**3.2**	28 min
IS-LSTM with Parameter Transfer	**11.63**±**1.6**	**15.64**±**2.5**	37 min	**14.24**±**2.1**	**21.81**±**3.2**	23 min

*Notes.* The bold values are significantly lower (p≤ 0.01) than non-bolded values in the same column.

**TABLE VI:** Mean RMSE±SD (mg/dL) for postprandial glucose prediction with 30- and 60- minute PH on the OpenAPS dataset

Method	Univariate		Multivariate	
	PH = 30 min	PH = 60 min	PH = 30 min	PH = 60 min
Single LSTM (Hameed and Kleinberg, 2020) [12]	18.89±2.9	31.63±3.8	19.92±3.1	32.74±4.0
CNN-LSTM (Jaloli and Cescon, 2022) [13]	19.12±3.2	30.83±3.7	19.56±3.2	31.79±3.8
Stacked LSTM (Rabby et al, 2021) [33]	15.98±2.7	22.48±3.3	16.36±2.8	22.92±3.5
IS-LSTM without Parameter Transfer	**11.54**±**1.6**	**17.61**±**2.3**	**12.07**±**1.7**	**18.26**±**2.4**
IS-LSTM with Parameter Transfer	**11.49**±**1.6**	**17.85**±**2.3**	**11.72**±**1.8**	**18.15**±**2.5**

*Notes.* The bold values are significantly lower (p≤ 0.01) than non-bolded values in the same column.

**TABLE VII:** Mean RMSE of univariate IS-LSTM with parameter transfer (30-minute PH) across varied initial days.

Initial days	OpenAPS		Replace-BG	
	High GV	Low GV	High GV	High GV
2	12.64±1.8	10.85±1.4*	15.28±2.3	13.75±1.9*
3	12.48±1.9	10.32±1.6*	15.34±2.3	13.62±1.9*
4	12.31±1.8	10.27±1.7*	14.96±2.1	13.49±1.8*
5	12.17±2.0	10.38±1.4*	14.45±2.1	13.50±1.8*
7	10.62±1.6	10.45±1.2	14.10±2.0	13.38±1.7*
8	10.74±1.7	10.49±1.1	13.81±2.0	13.18±1.6
10	10.53±1.5	10.14±1.3	13.63±1.8	13.15±1.6

*Notes.* A value marked by an asterisk * is statistically significantly lower than the value in the other column within the same dataset (p≤ 0.05).
